# Effect of age, sex, and county on postmortem findings in goats and sheep in Tennessee (USA), 2017–2021

**DOI:** 10.1371/journal.pone.0315680

**Published:** 2024-12-13

**Authors:** Andrea Lear, Wesley Sheley, Jessy Shanks, Brian Whitlock, Chika Okafor

**Affiliations:** 1 Department of Large Animal Clinical Sciences, College of Veterinary Medicine, University of Tennessee, Knoxville, Tennessee, United States of America; 2 Department of Biomedical and Diagnostic Sciences, College of Veterinary Medicine, University of Tennessee, Knoxville, Tennessee, United States of America; 3 Department of Animal Science, Herbert College of Agriculture, University of Tennessee, Knoxville, Tennessee, United States of America; GLA University, INDIA

## Abstract

Small ruminants, including sheep and goats, play an important role in the economy of American agriculture. They are susceptible to a variety of diseases that impact animal welfare and production. This study evaluated postmortem data from two different diagnostic laboratories in the state of Tennessee to discern common causes of death of small ruminants that were brought in for necropsy between 2017 to 2021. Data were prepared for analysis by selecting the predominant conditions observed at postmortem examination and risk factors including sex, age, season, and region were included in analysis. The predominant condition seen in both small ruminant species was endoparasitism. In both sheep and goats, female juvenile animals were more likely to be diagnosed with this condition at necropsy during summer months. Abortive diseases were the next most prominent condition diagnosed in both small ruminant species. The majority of these cases were due to an unknown cause and age was a significant risk factor in both sheep and goats. Neurological disorders in goats and pneumonia in sheep were included in the most prevalent diagnoses at postmortem examination with age being a significant risk factor. These findings suggest that many small ruminant deaths are attributed to infectious diseases that have herdwide implications. Producer education could be beneficial to help identify and implement control measures in a timely manner to help minimize production loss associated with common diseases.

## Introduction

Small ruminants, which include sheep and goats, have played a significant role in American agriculture for centuries. They are a versatile and valuable source of meat, milk, fiber, and even hide. According to the United States Department of Agriculture, approximately 5.2 million sheep and 2.66 million goats are kept in the United States. Small ruminants are particularly important in rural and pastoral communities where they provide essential livelihoods and contribute to local economies [1]. In addition to their economic importance, small ruminants are also valued for their environmental benefits, as they are often used for weed and brush control, reducing the risk of wildfires [2]. Beyond the many economic, environmental, and social benefits of small ruminant production, challenges are faced by producers in maintaining the health and welfare of their animals.

Small ruminants are susceptible to a range of diseases that can cause significant economic losses and pose a threat to human health [3]. One of the most common diseases affecting small ruminants in the United States is endoparasitism [4]. Effective disease prevention and management strategies are essential for maintaining the health and productivity of small ruminant populations, as well as ensuring the safety of the food products derived from these animals.

Anecdotally, Tennessee small ruminant farmers are uncertain on the most prevalent causes of death in their herds and where their control priorities could focus. The purpose of this study is to provide insight into common diseases discovered at postmortem examination of goats and sheep. The objective is to determine risk factors associated with predominant diseases in small ruminants in the state of Tennessee. Ultimately, this manuscript aims to emphasize the need for disease prevention and control, and highlight areas of continued research and innovation in this area. Results from this would be useful for Tennessee extension officers to educate small ruminant farmers on measures that could improve their herd health.

## Methods

### Ethics statement

Ethical approval was not required for this study as this is a retrospective study of animal carcasses submitted for necropsy to the C.E. Kord Animal Health Diagnostic Laboratory (KORD) and the University of Tennessee, College of Veterinary Medicine (UTCVM) veterinary diagnostic laboratories.

### Data source, retrieval, and preparation

Our study population was all small ruminants (goats and sheep) necropsied between 2017 and 2021 and whose owners resided in the state of Tennessee. Necropsy reports of all goats and sheep from 2017 to 2021 were obtained from both the C.E. Kord Animal Health Diagnostic Laboratory (KORD) and the University of Tennessee, College of Veterinary Medicine (UTCVM) veterinary diagnostic laboratories. Both KORD and UTCVM are the state veterinary diagnostic laboratories for Tennessee and provide free laboratory testing for food and fiber animals, including small ruminants, for residents. The producer pays for the cost of body disposal which is $0.23 per pound of body weight. The state covers the cost of necropsy ($200) as well as an additional $300 per animal that can be used for ancillary diagnostic testing as indicated.

The requested variables during data retrieval were that ruminant owners must reside in Tennessee and the following information about each goat and sheep necropsied at the labs: county of residence for the owner of the animal being necropsied, date of necropsy, age, sex, breed, and diagnoses reported. Next, the diagnoses reported, often containing text-form responses, were reviewed by a board-certified veterinary anatomic pathologist (WS) and separated into primary, secondary, and tertiary based on frequency of the diagnosis in that species. Each primary diagnosis was assigned to a representative body system. Diagnoses directly related to another diagnosis listed were removed. For example, in goats with endoparasitism including *Haemonchus* sp., a diagnosis of anemia was removed as *Haemonchus* infection is a direct cause of anemia. Where applicable, the specific organisms or parasites contributing to the most common conditions were captured. Subsequently, the obtained data was transferred from Microsoft Excel to SAS 9.4 software (**SAS** Institute Inc) for data validation and analysis.

### Data preparation and variables

First, the conditions found at necropsy in these small ruminants were summarized, revealing the most common conditions. Afterwards, other conditions were collectively categorized as controls for the purpose of identifying factors associated with the occurrence of the most common condition. Where applicable, the specific organisms or parasites contributing to the most common conditions were summarily presented. Hence, the primary variable of interest utilized in selecting the final dataset for analysis was the predominant condition at death in each of the small ruminants. Other selected variables for analysis in each dataset included sex, age, breed, primary purpose of animal’s breed, year, season (quarter of year), county, and region of the state (East, Middle, and West Tennessee). Specifically, the "month" variable was categorized into the following quarters: Quarter 1 (Winter; January, February, and March), Quarter 2 (Spring; April, May, and June), Quarter 3 (Summer; July, August, and September), and Quarter 4 (Fall; October, November, and December). Assignment of counties into regions of Tennessee was done based on the Grand Divisions of Tennessee. Animals were categorized according to the following ages: fetus (less than 2 days old), neonate (3 days to a month old), juvenile (above 1 month to 6 months old), and adult (above 6 months old). The top prevalence breeds were reported, and fewer common breeds were aggregated to “other” according to the primary purpose of the breed (meat, dairy, or mixed).

### Statistical analysis

First, descriptive statistics (counts and percentages) were used to summarize the frequency and distributions of obtained diseased conditions as well as signalment, and geographical and temporal demographic attributes of animals. Emphasis was placed on the most common conditions in each species of small ruminant. Next, the association between the most common condition at death and the explanatory variables (the signalment, geographical, and temporal attributes of the animal) was analyzed. Goat and sheep population data for each county in Tennessee were obtained from the 2017 census of the National Agricultural Statistical Service [5]. Choropleth maps were created to display data in a visually concise format based on the state-wide diagnostic laboratory data on the most common condition at death in small ruminants, and 2017 census of the National Agricultural Statistical Service using ArcGIS 10.5 (ESRI, Redlands, CA).

Both univariable and multivariable logistic regression analyses were performed to test the associations between the explanatory variables and the most common conditions at death for goats and sheep, accordingly. These data analyses were conducted in SAS9.4 for windows 64x (Cary, NC). Odds ratios and their confidence intervals (CIs) were used to measure the strength of associations between the explanatory variables and the outcome. A *P* value of ≤ 0.05 was considered significant. In fitting the final multivariable logistic model, all the variables in the univariable analyses were examined and interactions between selected variables were tested. The tested interactions were sex and breed; age and sex; age and breed; and year and quarter of the year. We accessed for confounding in the model building process. A variable was classified as a confounder if it changed the coefficient of a previously significant variable in the logarithm scale by at least 20% [6]. All confounders would be included in the final model but with specific mention that they are confounders. The overall assessment of the final model was done using the Bayesian Information.

## Results

**Fig 1:**
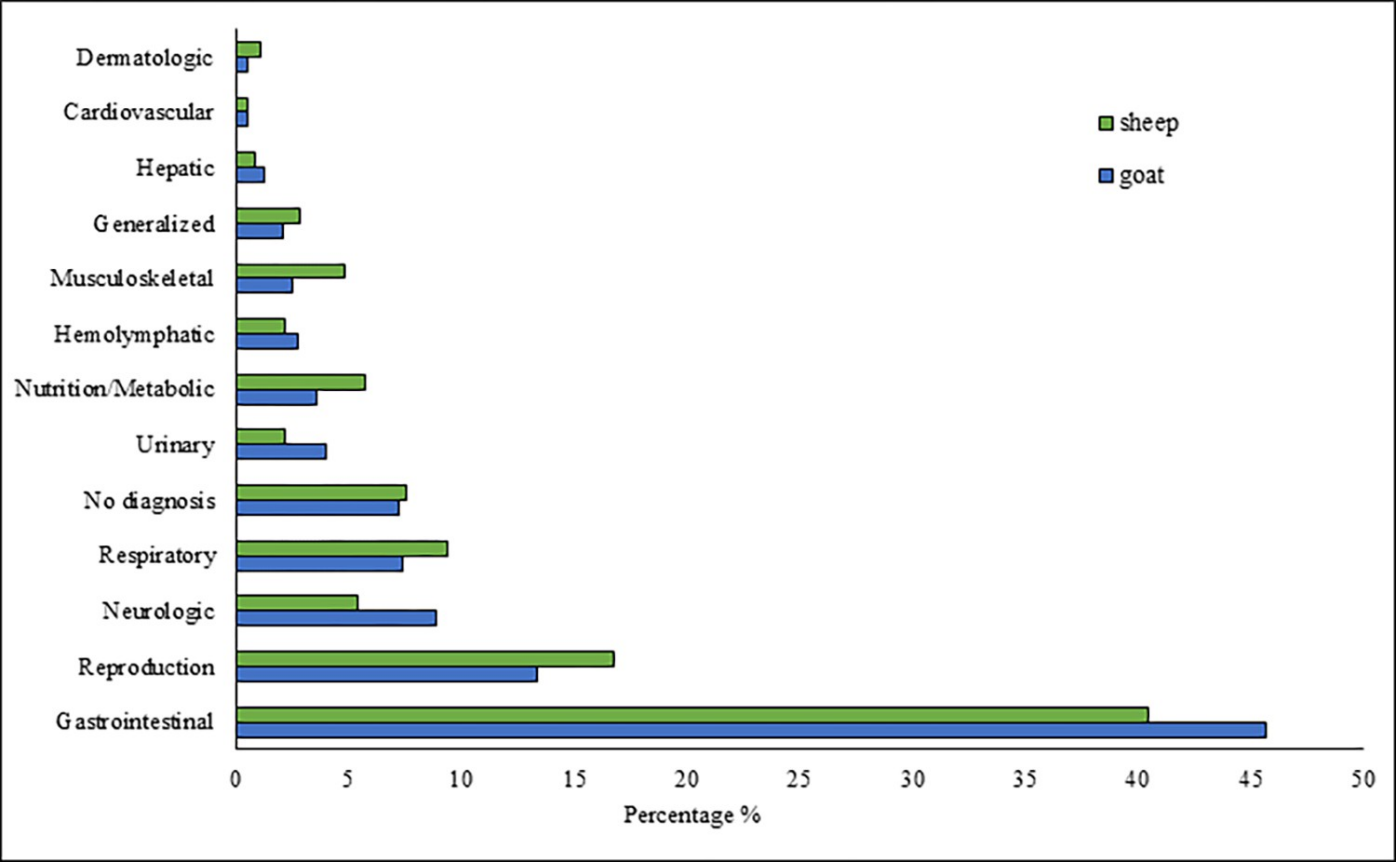
Distribution of the body systems with pathologic lesions in the assessment of the effect of age, sex, and county on postmortem findings in goats and sheep in Tennessee (USA), 2017–2021. Total number (n) for goats is 1243 and sheep is 554.

There were 1808 observations in the original dataset. Of these, 11 observations whose counties not located in the state of Tennessee were deleted. Hence, 1797 observations, 1243 goats and 554 sheep records, were used in further analysis. The distribution of the body systems affected based on postmortem diagnosis is presented in Fig 1 and Table 1 for goats and sheep. Endoparasitism was the most common diagnosis at necropsy for small ruminants (Table 2). For both goats and sheep, abortion, neurologic disease and pneumonia were among the top 5 most numerous conditions found (Table 2). A complete list of primary diagnoses at postmortem examination for both goats (S1 Table) and sheep (S2 Table) is provided in supplemental documents.

**Table 1:** Distribution of the body systems with pathologic lesions in the assessment of the effect of age, sex, and county on postmortem findings in goats and sheep in Tennessee (USA), 2017–2021.

Lesioned Body System	Goats count (%)	Sheep count (%)
Gastrointestinal	568 (45.70)	224 (40.43)
Reproduction	166 (13.35)	93 (16.79)
Neurologic	111 (8.93)	30 (5.42)
Respiratory	92 (7.40)	52 (9.39)
Unknown/No diagnosis	90 (7.24)	42 (7.58)
Urinary	50 (4.02)	12 (2.17)
Nutrition/Metabolic	45 (3.62)	32 (5.78)
Hemolymphatic	34 (2.74)	12 (2.17)
Musculoskeletal	31 (2.49)	27 (4.87)
Generalized	26 (2.09)	16 (2.89)
Hepatic	16 (1.29)	5 (0.90)
Cardiovascular	7 (0.56)	3 (0.54)
Dermatologic	7 (0.56)	6 (1.08)
**Total**	**1243 (100%)**	**554 (100%)**

**Table 2:** Distribution of top 5 conditions identified in the assessment of the effect of age, sex, and county on postmortem findings in goats and sheep in Tennessee (USA), 2017–2021.

Top 5 conditions at death	Goats count (%)	Sheep count (%)
Endoparasitism	497 (39.98)	182 (32.85)
Abortion	133 (10.7)	77 (13.90)
Neurologic disease	111 (8.93)	30 (5.42)
Pneumonia	83 (6.68)	42 (7.58)
Intestinal disease	49 (3.94)	*
Trauma	*	20 (3.61)
No diagnosis	90 (7.24)	42 (7.58)
Others	280 (22.53)	161 (29.06)

*included in the others category.

### Most common conditions in goats

**Fig 2:**
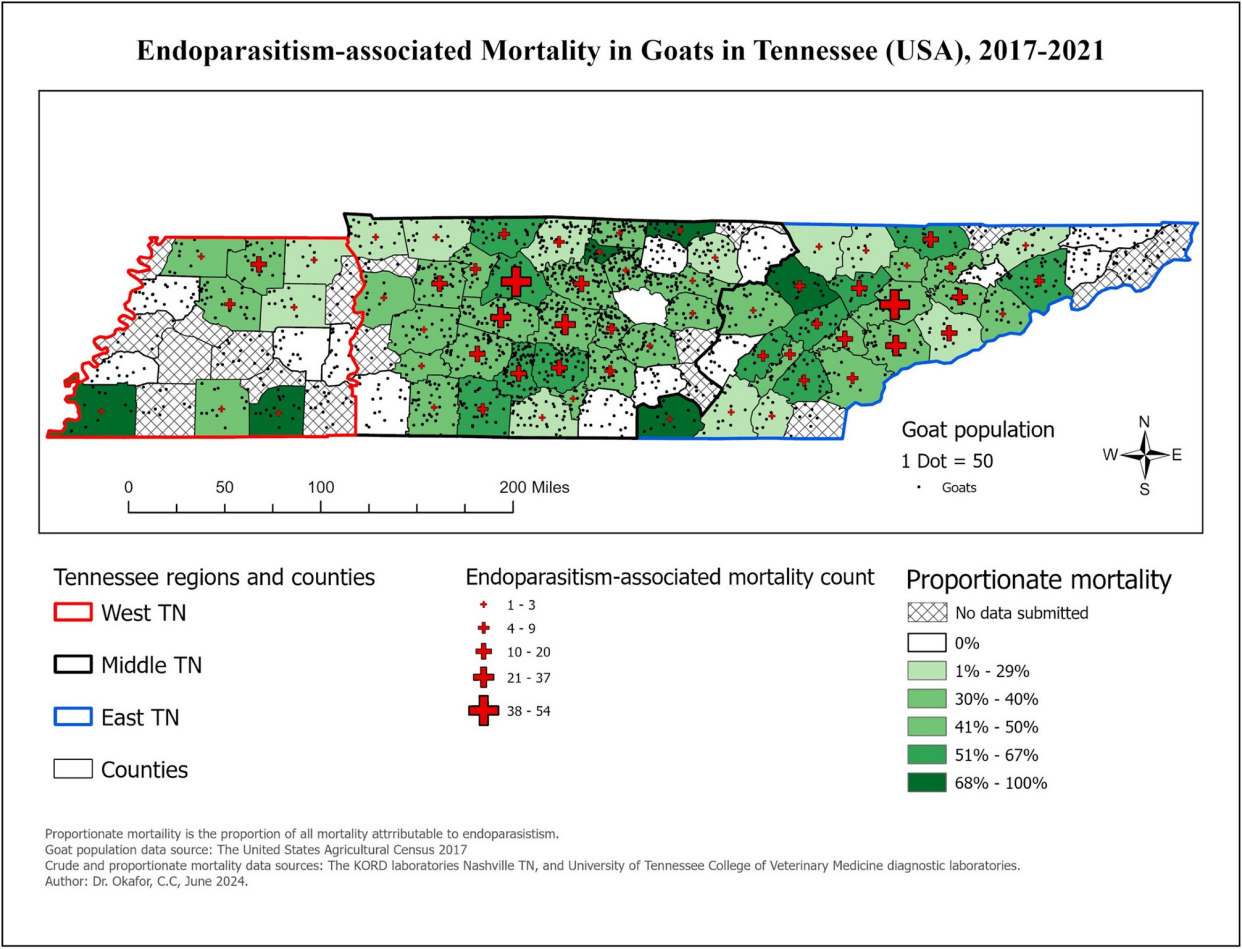
Choropleth map showing the distribution of goats diagnosed with endoparasitism at time of necropsy in Tennessee, 2017–2021.

By subsequent classification of 1,243 goats, endoparasitism was the primary diagnosis in 497 cases (39.98%). Choropleth maps showing the distribution of mortality from endoparasitism in goats is presented in Fig 2.

The top three endoparasite genera found in goats were *Haemonchus* sp. (n = 230), coccidia (n = 109), and strongyles (n = 18). The distribution of the explanatory variables associated with endoparasitism in goats is presented in Table 3. In the final fitted multivariable model (Table 4), season, age, and sex of goats were significantly associated with the odds of endoparasitism in goats at the time of necropsy. Controlling for the effect of season; however, there was a significant interaction between age and sex of goats. In this interaction, the odds of endoparasitism in juveniles compared to fetuses were over 9 times higher in females but 6 times higher in males. Accounting for this interaction, in comparison to spring, the odds of the outcome were 2.11 and 2.6 times higher in fall and summer, respectively. Also, the odds of the outcome were 2 times higher in summer in comparison to winter.

**Table 3:** Logistic univariable analysis for associations between various factors and endoparasitism in Tennessee goats, 2017–2021.

Variable	Category	Endoparasitism N (%)	No Endoparasitism N (%)	OR	95% CI	*P* value
Age	Adult	305 (45.05)	372 (54.95)	Ref	—	—
	Fetus	34 (12.55)	237 (87.45)	0.18	0.11–0.28	<0.0001
	Neonate	16 (36.36)	28 (63.64)	0.70	0.32–1.51	0.5951
	Juvenile	142 (56.57)	109 (43.43)	1.59	1.11–2.27	0.0057
Sex	Male	191 (41.79)	266 (58.21)	Ref	—	—
	Female	291 (42.48)	394 (57.52)	1.03	0.78–1.35	0.9657
	Unknown	15 (14.85)	86 (85.15)	0.24	0.13–0.47	<0.0001
Breed	Nubian	49 (46.67)	56 (53.33)	Ref	—	—
	Boer	73 (40.11)	109 (59.89)	0.77	0.40–1.46	0.8000
	Mixed	59 (33.71)	116 (66.29)	0.58	0.30–1.12	0.1537
	Pygmy	43 (36.44)	75 (63.56)	0.66	0.32–1.34	0.4666
	Nigerian dwarf	37 (34.58)	70 (65.42)	0.60	0.29–1.26	0.3143
	Other Dairy	31 (40.79)	45 (59.21)	0.79	0.36–1.74	0.9468
	Other Meat	14 (58.33)	10 (41.67)	1.60	0.49–5.28	0.8343
	Not reported	191 (41.89)	265 (58.11)	0.82	0.47–1.45	0.9055
Test laboratory	Kord	299 (41.36)	424 (58.64)	Ref	—	—
	UTCVM	198 (38.08)	322 (61.92)	0.87	0.69–1.10	0.2446
Year	2017	106 (43.98)	135 (56.02)	Ref	—	—
	2018	95 (39.42)	146 (60.58)	0.83	0.53–1.30	0.6997
	2019	93 (37.35)	156 (62.65)	0.76	0.48–1.19	0.3721
	2020	113 (40.36)	167 (59.64)	0.86	0.56–1.33	0.8189
	2021	90 (38.79)	142 (61.21)	0.81	0.51–1.28	0.6071
Month	January	46 (29.11)	112 (70.89)	Ref	—	—
	February	35 (25.00)	105 (75.00)	0.81	0.39–1.68	0.9923
	March	40 (27.21)	107 (72.79)	0.91	0.45–1.85	1.0000
	April	23 (24.47)	71 (75.53)	0.79	0.35–1.80	0.9921
	May	34 (38.20)	55 (61.80)	1.51	0.69–3.28	0.7087
	June	55 (57.89)	40 (42.11)	3.35	1.57–7.13	0.0001
	July	50 (55.56)	40 (44.44)	3.04	1.42–6.54	0.0006
	August	49 (58.33)	35 (41.67)	3.41	1.56–7.47	0.0002
	September	43 (57.33)	32 (42.67)	3.27	1.45–7.37	0.0006
	October	41 (53.95)	35 (46.05)	2.85	1.28–6.38	0.0031
	November	37 (40.66)	54 (59.34)	1.67	0.77–3.60	0.4108
	December	44 (42.31)	60 (57.69)	1.79	0.86–3.73	0.2162
Season	Spring	97 (29.39)	233 (70.61)	Ref	—	—
	Summer	154 (57.25)	115 (42.75)	3.22	2.14–4.83	<0.0001
	Fall	121 (50.00)	121 (50.00)	2.40	1.59–3.64	<0.0001
	Winter	125 (31.09)	277 (68.91)	1.08	0.74–1.59	0.9253
Region (State)	West TN	29 (34.94)	54 (65.06)	Ref	—	—
	East TN	202 (38.19)	327 (61.81)	1.15	0.68–1.94	0.7034
	Middle TN	266 (42.16)	365 (57.84)	1.36	0.81–2.28	0.2819

**Table 4:** Final multivariable logistic regression analysis of factors associated with endoparasitism in Tennessee goats, 2017–2021.

Variable	Category	OR	95% CI	*P* value
Age	Juvenile vs. Adult	62.50	<0.01—∞	1.0000
	Neonate vs. Adult	90.91	<0.01—∞	1.0000
	Fetus vs. Adult	15.63	<0.01—∞	1.0000
	Juvenile vs Neonate	0.68	0.20–2.30	0.8492
	Fetus vs. Neonate	0.17	0.06–0.50	0.0002
	Juvenile vs Fetus	4.04	1.67–9.76	0.0003
Sex	Female vs. Male	1.43	0.84–2.44	0.2519
	Unknown vs. Male	0.04	<0.01—∞	0.9994
	Unknown vs. Female	0.03	<0.01—∞	0.9992
SexXAge	Among Males, Juvenile vs. Adult	1.12	0.54–2.35	1.000
	Among Males, Neonate vs. Adult	0.41	0.07–2.35	0.8772
	Among Males, Fetus vs. Adult	0.19	0.06–0.62	0.0004
	Among Males Juvenile vs. Neonate	2.76	0.46–16.50	0.7821
	Among Males, Fetus vs. Neonate	0.46	0.06–3.48	0.9839
	Among Males, Juvenile vs. Fetus	6.03	1.70–21.41	0.0002
	Among Females, Juvenile vs. Adult	2.05	0.94–4.46	0.1009
	Among Females, Neonate vs. Adult	1.10	0.20–6.13	1.000
	Among Females, Fetus vs. Adult	0.22	0.08–0.58	<0.0001
	Among Females, Juvenile vs. Neonate	1.86	0.30–11.62	0.9942
	Among Females, Fetus vs. Neonate	0.20	0.03–1.36	0.2027
	Among Females, Juvenile vs. Fetus	9.36	2.92–30.01	<0.0001
	Among Unknowns, Juvenile vs. Adult	∞	<0.01—∞	1.000
	Among Unknowns, Neonate vs. Adult	∞	<0.01—∞	1.000
	Among Unknowns, Fetus vs. Adult	∞	<0.01—∞	1.000
	Among Unknowns, Juvenile vs. Neonate	0.06	<0.01–2.91	0.4286
	Among Unknowns, Fetus vs. Neonate	0.05	<0.01–1.14	0.075
	Among Unknowns, Juvenile vs. Fetus	1.17	0.07–20.12	1.000
Season	Fall vs. Spring	2.11	1.31–3.40	0.0004
	Summer vs. Spring	2.60	1.62–4.17	<0.0001
	Winter vs. Spring	1.25	0.80–1.95	0.5563
	Fall vs. Winter	1.68	1.06–2.66	0.0189
	Summer vs. Winter	2.07	1.31–3.29	0.0003
	Fall vs. Summer	0.81	0.50–1.32	0.6821

Abortion was the second most frequent condition, and of the 133 goats diagnosed, most were of unknown cause (n = 112). Specifically, viral (n = 7), bacterial (n = 9), protozoal (n = 2), toxic (n = 2), and mycotic (n = 1) agents were identified only in 21 cases presented for abortion. All viral agents (n = 7) were bovine viral diarrhea virus (BVDV). Season (p < 0.01), region (p = 0.02), and breed purpose (p = 0.01) were significant risk factors associated with abortion in goats (Table 5). Neurologic disease was diagnosed in 111 goats, representing 8.93% of total goats. Predominant conditions in these animals included unspecified bacterial neurologic conditions (n = 56), meningoencephalitis (n = 20), and listeriosis (n = 3). Age (p = 0.0007), and region (p = 0.0133) were significant risk factors associated with neurological diseases in goats (Table 6).

**Table 5:** Final multivariable logistic regression analysis of factors associated with abortion in Tennessee goats, 2017–2021.

Variable	Category	OR	95% CI	*P* value
Season	Fall vs. Summer	7.06	1.77–28.24	0.0041
	Spring vs. Summer	11.42	3.02–43.17	0.0001
	Winter vs. Summer	23.73	6.46–87.19	<0.0001
Region (State)	East vs. West	6.94	1.51–31.93	0.0119
	Middle vs. West	5.23	1.15–23.77	0.0314
Purpose	Dairy vs. Meat	1.05	0.50–2.19	0.9996
	Fiber vs. Meat	<0.01	<0.01—∞	1.0000
	N/A vs. Meat	2.17	1.16–4.07	0.0092
	Unknown vs. Meat	1.20	0.52–2.76	0.9616

**Table 6:** Final multivariable logistic regression analysis of factors associated with neurological diseases in Tennessee goats, 2017–2021.

Variable	Category	OR	95% CI	*P* value
Age	Juvenile vs. Adult	0.36	0.16–0.83	0.0089
	Neonate vs. Adult	0.70	0.17–2.79	0.9070
	Fetus vs. Adult	0.39	0.18–0.85	0.0105
	Juvenile vs Neonate	0.52	0.40–9.09	0.7061
	Fetus vs. Neonate	0.57	0.12–2.61	0.7742
	Fetus vs Juvenile	1.08	0.38–3.07	0.9972
Region (State)	Middle vs. East	1.87	1.12–3.12	0.0112
	West vs. East	1.13	0.35–3.66	0.9659
	Middle vs. West	1.65	0.53–5.13	0.5538

### Most common conditions in sheep

**Fig 3:**
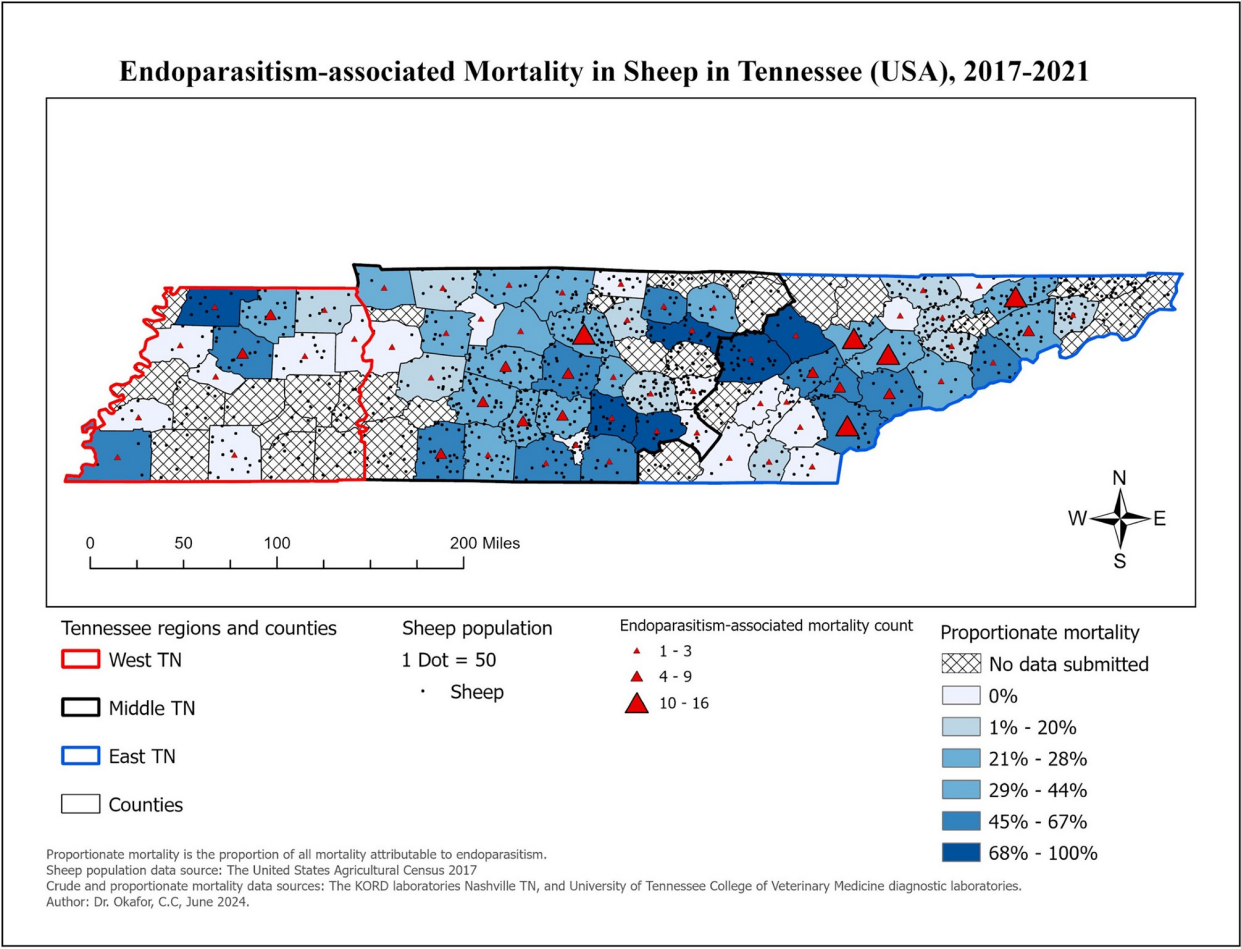
Choropleth maps showing the distribution of sheep diagnosed with endoparasitism at time of necropsy in Tennessee, 2017–2021.

Similarly, 182 of the 554 (32.85%) sheep had a diagnosis of endoparasitism. The top three predominant endoparasite genera found in sheep were *Haemonchus* sp. (n = 73), coccidia (n = 26), and strongyles (n = 7). The distribution of the explanatory variables associated with endoparasitism in sheep is presented in Table 7. Choropleth maps showing the distribution of sheep diagnosed with endoparasitism at time of necropsy is presented in Fig 3.

**Table 7:** Logistic univariable analysis for associations between various factors and endoparasitism in Tennessee sheep, 2017–2021.

Variable	Category	Endoparasitism N (%)	No Endoparasitism N (%)	OR	95% CI	*P* value
Age	Adult	75 (30.24)	173 (69.76)	Ref	—	—
	Fetus	6 (4.88)	117 (95.12)	0.12	0.04–0.34	<0.0001
	Neonate	4 (10.81)	33 (89.19)	0.28	0.08–1.04	0.0589
	Juvenile	97 (66.44)	49 (33.56)	4.57	2.68–7.79	<0.0001
Sex	Male	64 (34.97)	119 (65.03)	Ref	—	—
	Female	114 (35.08)	211 (64.92)	1.01	0.65–1.55	0.9996
	Unknown	4 (8.70)	42 (91.30)	0.18	0.05–0.60	0.0032
Breed	Hampshire	8 (23.53)	26 (76.47)	Ref	—	—
	Katahdin	57 (39.58)	86 (60.42)	2.13	0.73–6.24	0.2467
	Mixed	27 (31.76)	58 (68.24)	1.51	0.48–4.75	0.7741
	N/A	74 (32.89)	151 (67.11)	1.59	0.56–4.55	0.6352
	Other Meat	14 (23.73)	45 (76.27)	1.01	0.29–3.51	1.000
	Other Dairy/fiber	2 (28.57)	5 (71.43)	1.30	0.13–12.68	0.9970
Test laboratory	Kord	90 (31.47)	196 (68.53)	Ref	—	—
	UTCVM	92 (34.33)	176 (65.67)	1.14	0.80–1.62	0.4743
Year	2017	51 (39.84)	77 (60.16)	Ref	—	—
	2018	46 (42.59)	62 (57.41)	1.12	0.58–2.16	0.9829
	2019	23 (21.10)	86 (78.90)	0.40	0.20–0.84	0.0088
	2020	27 (24.77)	82 (75.23)	0.50	0.25–1.01	0.0531
	2021	35 (35.00)	65 (65.00)	0.81	0.41–1.61	0.8832
Month	January	18 (27.69)	47 (72.31)	Ref	—	—
	February	12 (16.44)	61 (83.56)	0.51	0.16–1.66	0.6202
	March	10 (12.99)	67 (87.01)	0.39	0.12–1.32	0.2388
	April	7 (18.42)	31 (81.58)	0.59	0.15–2.39	0.9464
	May	14 (37.84)	23 (62.16)	1.59	0.47–5.40	0.9446
	June	22 (62.86)	13 (37.14)	4.42	1.27–15.36	0.0094
	July	38 (66.67)	19 (33.33)	5.22	1.74–15.71	0.0004
	August	11 (32.35)	23 67.65)	1.25	0.35–4.50	0.9999
	September	17 (40.48)	25 (59.52)	1.78	0.55–5.72	0.7821
	October	12 (40.00)	18 (60.00)	1.74	0.48–6.36	0.8885
	November	12 (38.71)	19 (61.29)	1.65	0.46–5.98	0.9355
	December	9 (25.71)	26 (74.29)	0.90	0.24–3.41	1.0000
Season	Spring	31 (20.39)	121 (79.61)	Ref	—	—
	Summer	71 (56.35)	55 (43.65)	5.04	2.67–9.50	<0.0001
	Fall	41 (39.81)	62 (60.19)	2.58	1.32–5.04	0.0026
	Winter	39 (22.54)	134 (77.46)	1.14	0.60–2.15	0.9334
Region (State)	West TN	19 (35.19)	35 (64.81)	Ref	—	—
	East TN	91 (33.96)	177 (66.04)	0.95	0.48–1.86	0.9677
	Middle TN	72 (31.03)	160 (68.97)	0.83	0.42–1.65	0.7151

In the final fitted multivariable model (Table 8), both “Season” (p = 0.0044) and “Age” (p <0.0001) were significantly associated with presence of endoparasitism at death in sheep. The results indicate a significantly increased prevalence in juveniles compared to all other ages (p < 0.001). The odds of endoparasitism in juvenile sheep were almost 5, 14, and 35 times as high in comparison to adults, neonates, and fetuses, respectively. No significant difference (p = 0.2372) was identified in the odds of the outcome between neonates and adults or fetuses. The odds of the outcome were almost 3 times as high in summer in comparison to spring and this was statistically significant (p = 0.0021).

**Table 8:** Final multivariable logistic regression analysis of factors associated with endoparasitism in Tennessee sheep, 2017–2021.

Variable	Category	OR	95% CI	*P* value
Age	Juvenile vs. Adult	4.74	2.56–8.78	<0.0001
	Neonate vs. Adult	0.35	0.08–1.48	0.2372
	Fetus vs. Adult	0.14	0.04–0.43	<0.0001
	Juvenile vs Neonate	13.59	3.05–60.61	<0.0001
	Fetus vs. Neonate	0.39	0.07–2.24	0.5050
	Juvenile vs Fetus	34.99	10.13–120.92	<0.0001
Season	Fall vs. Spring	1.86	0.82–4.25	0.2128
	Summer vs. Spring	2.97	1.36–6.49	0.0021
	Winter vs. Spring	2.16	0.95–4.88	0.0749
	Fall vs. Winter	0.86	0.39–1.93	0.9658
	Summer vs. Winter	1.38	0.64–2.98	0.7067
	Fall vs. Summer	0.63	0.29–1.34	0.3914

Abortion was the second most common condition, and of the 77 sheep diagnosed, most were of unknown cause (n = 54). Specific pathogens identified included bovine viral diarrhea virus (BVDV, n = 6), and cache valley fever virus (CVFV, n = 3). Only age of sheep was significantly associated with abortion (p < 0.0001). Pneumonia was observed in 7.58% of sheep cases (n = 42). Of the 42 cases, 33 were caused by bacterial organisms. The predominant bacteria was *Mannheimia haemolytica* and less common ones were *Pastuerella multocida* and *Mycoplasma* spp. Age was the only factor significantly associated with pneumonia diagnosis; where the odds of diagnosis of pneumonia in neonates were 4 times more likely than in juveniles (95% CI 1.35–12; p = 0.0125). Whereas the odds of diagnosis of pneumonia in neonates were approximately 5 times higher than in fetuses (95% CI 1.42–15; p = 0.0109).

## Discussion

Small ruminants are an important part of the economy in the state of Tennessee. As of January 1, 2024, the Tennessee sheep inventory was estimated at 51,000 head, goats were estimated at 72,000 head by the National Agricultural Statistical Service [7]. According to the 2017 Census of Agriculture the value of sheep and goats sold in Tennessee was estimated to be $5,423,000, and $5,598,000, respectively [5]. Sheep and goats are also an important part of the economy nationwide with sheep and lambs valued at $686,371,000 and all goats were valued at $163,648,000 nationwide in 2017 [8].

The objective of this report is to characterize the causes of morbidity and mortality in small ruminants presented for postmortem examination in the state of Tennessee over a 5-year period from 2017–2021. The findings from this study provide valuable insights which can significantly impact future interventions and policies for livestock management in the region. Given the economic importance of small ruminants in Tennessee, these findings highlight critical areas for improvement in herd health management and disease prevention.

Overall, we found that endoparasitism, abortion, neurologic diseases, and pneumonia were the most frequently diagnosed conditions in these animals. Of these conditions, endoparasitism was by far the most common. Age, season, and sex were significant variables of increased odds of endoparasitism. Increased seasonal risks were expected as warm, humid climate accelerates the development and reproduction of the parasites [9]. Younger animals were found to be at greater risk of developing endoparasitism in our current study and in other reports [10]. This may be associated with unique physiologic stressors in young stock [11,12]. Furthermore, younger animals are considered to be more immunologically naive [13,14], resulting in increased risk of infectious disease. Surprisingly, breed was not a significant variable associated with endoparasitism. Genetic variation between breeds has been determined to play an important role in susceptibility and resistance to internal parasites in both sheep and goats [15,16]. In our study population, young females were more likely to develop endoparasitism which is in consonance with the findings of a previous study [10]. Sex of animals and reproductive status, most notably pregnancy, affect the immune system [11,17]. In small ruminants, immune relaxation during gestation is a documented phenomenon resulting in increased susceptibility to endoparasitism [17]. Due to the retrospective nature of our data set, reproductive status of the animals was not always recorded. Therefore, it is unclear how the reproductive status of young, female small ruminants is associated with endoparasitism at necropsy.

Many factors, including production status, plane of nutrition, and grazing practices affect the risk of parasites in livestock [18]. As endoparasitism was identified as the most prevalent issue, this suggests a need for enhanced parasite management strategies in the state. This may involve encouraging producers to adopt sustainable and integrated parasite control practices, such as the use of the FAMACHA system [19], and alternative preventions or therapies [20], like copper boluses, to reduce the reliance on anthelmintics and combat parasite resistance [21]. Strategic and sustainable parasite management relies on frequent monitoring and identification of high-risk individuals [22]. Producer education level [10] and perception [23,24] are highly relevant when understanding endoparasitism risk and implementing control practices. Extension agents and veterinarians should focus on educating producers about the risks posed by endoparasites, especially in young animals and pregnant females, who are more vulnerable. Providing training on parasite monitoring and management techniques could be a crucial step in reducing mortality and improving the overall health of small ruminant populations.

Abortion was the second most common condition identified in both sheep and goats. Many different pathogens including an array of viruses, bacteria, and protozoa are important causes of infectious abortion in small ruminant herds [25]. Although specific pathogens were identified, unfortunately, for the vast majority of abortion cases, a specific cause was undetermined. Failing to identify a cause of abortion in small ruminants is not uncommon with one study reporting a diagnostic rate of only 42.2% when performing a full bacterial and fungal diagnostic panel [26]. Major limitations to identifying the etiology of an abortion include lack of appropriate sample submission (*i*.*e*., no placenta included, fetus and placenta markedly autolyzed, *etc*.) and financial limitations as full abortion diagnostic panels can be cost restrictive.

In terms of abortion management, the study underscores the importance of accurate diagnostics to determine the causes of infectious abortions. Promoting better sample submission practices, including ensuring proper fetal and placental samples for diagnostics, and exploring more cost-effective options for producers could improve diagnostic rates and lead to more effective disease control. Veterinary practitioners and extension agents could work with producers to establish protocols for proper sample collection and explore financial assistance or subsidies for diagnostic testing to mitigate the economic losses associated with undiagnosed abortions.

Our results demonstrate that infectious diseases are a predominant finding at postmortem examination of sheep and goats. These results highlight the importance of early identification of infectious diseases and quick institution of control measures in the state’s, and possibly the region’s, small ruminant industry. Increased efforts in disease surveillance, vaccination programs, and biosecurity measures could be beneficial. State agencies might consider allocating more resources toward supporting diagnostic laboratories to enhance data collection, analysis, and reporting, which would improve disease tracking and management at both local and regional levels.

Due to the retrospective nature of this report, access to data was limited and information bias may exist. Selection bias is a usual limitation of laboratory studies because subjects in the study could have been influenced by factors such as comorbidities in the animals or the socio-economic status of their owners. Owners with higher socio-economic status may be more likely to send their animals to the hospital or to the diagnostic laboratories, making results of such studies not representative of animals in the general population. However, such selection bias could be minimal in the current study because all small ruminant owners in TN could submit their dead animals for necropsy at no cost. Possibly, farmers with relatively higher educational awareness could take advantage of the complementary necropsy services by the state. Although not evaluated in the current study, any such disparity in the educational knowledge of the farmers with respect to submitting animals for necropsy could have introduced a selection bias that affects the external validity or generalization of the current study. The effect of this potential bias would be non-differential because it is less likely that those farmers whose animals die from endoparasitism would be different from those whose animals died from other conditions. Any such non-differential misclassification bias would have tended towards the null. Should that have been the case, our observed odds ratios could have been diluted further than it should. Ultimately, the study’s findings can inform state/lab policies, guiding extension programs, producer training, and veterinary practices to reduce disease-related losses and improve livestock productivity in Tennessee’s small ruminant sector. Future retrospective studies would benefit from standardization in reporting diagnoses amongst state supported diagnostic laboratories, allowing for ease of analysis and interpretation of data sets.

## Conclusion

This study demonstrates the importance of frequent monitoring of small ruminant herds for common conditions allowing for quick control implementation. Producers are encouraged to work with their veterinary and extension experts in developing management plans to decrease morbidity and mortality related to infectious diseases. Further education and outreach measures should be taken to enhance producer’s knowledge to prevent outbreaks of disease in small ruminant herds. Future research questions could include investigating current on-farm management practices (or lack thereof) that are contributing to these small ruminant ailments, as well as evaluating producer knowledge and aiming to improve it. Additionally, this type of retrospective analysis can be performed on a regular basis in multiple food animal species for which necropsy data exists in the state of Tennessee in addition to small ruminants. Increasing educational opportunities, refining management practices, and continuing to monitor necropsy data from across the state could reduce small ruminant mortality through producer education.

## Supporting information

S1 Table Primary diagnoses found in goats during postmortem examination in Tennessee, 2017–2021. (PDF)

S2 Table Primary diagnoses found in sheep during postmortem examination in Tennessee, 2017–2021. (PDF)

S3 Table Raw data file for sheep and goat postmortem examinations in Tennessee, 2017–2021. (XLSX)
