# Supplementary material for: Effect of age, sex, and county on postmortem findings in goats and sheep in Tennessee (USA), 2017–2021
Source: PLoS One. 2024 Dec 13;19(12):e0315680. doi: 10.1371/journal.pone.0315680 (PMC11642948; doi:10.1371/journal.pone.0315680)
Supplement: S1 Table — (PDF) [file pone.0315680.s001.pdf]

S1 Table.

Primary Diagnoses in Goats				
Diagnosis	Frequency	Percent	Cumulative Frequency	Cumulative Percent
Endoparasitism	497	39.98	497	39.98
Abortion	133	10.70	630	50.68
Neurologic disease	111	8.93	741	59.61
No diagnosis	90	7.24	831	66.85
Pneumonia	83	6.68	914	73.53
Intestinal	49	3.94	963	77.47
Urolithiasis	30	2.41	993	79.89
Trauma	19	1.53	1012	81.42
Emaciation	14	1.13	1026	82.54
Forestomach	14	1.13	1040	83.67
Anemia	10	0.80	1050	84.47
Caseous lymphadenitis	10	0.80	1060	85.28
Sepsis	10	0.80	1070	86.08
Metritis	8	0.64	1078	86.73
Thymoma	8	0.64	1086	87.37
dystocia	8	0.64	1094	88.01
Heart failure	7	0.56	1101	88.58
Hepatic necrosis	7	0.56	1108	89.14
Pregnancy toxemia	7	0.56	1115	89.70
Copper toxicity	6	0.48	1121	90.19
Dystocia	6	0.48	1127	90.67
Mastitis	6	0.48	1133	91.15
Copper deficiency	5	0.40	1138	91.55
Peritonitis	5	0.40	1143	91.95
Hepatitis	4	0.32	1147	92.28
Kidney disease	4	0.32	1151	92.60

Primary Diagnoses in Goats				
Diagnosis	Frequency	Percent	Cumulative Frequency	Cumulative Percent
Bladder	3	0.24	1154	92.84
CAEV	3	0.24	1157	93.08
DJD	3	0.24	1160	93.32
Hepatic lipidosis	3	0.24	1163	93.56
Iron toxicity	3	0.24	1166	93.81
Polyarthritis	3	0.24	1169	94.05
Pulmonary edema	3	0.24	1172	94.29
Tetanus	3	0.24	1175	94.53
Abomasal disease	2	0.16	1177	94.69
BVDV	2	0.16	1179	94.85
Dermatitis	2	0.16	1181	95.01
Hemangiosarcoma	2	0.16	1183	95.17
Hepatocellular necrosis	2	0.16	1185	95.33
Lymphoma	2	0.16	1187	95.49
Omphalitis	2	0.16	1189	95.66
Pregnancy toxemia	2	0.16	1191	95.82
Pulmonary dysmaturity	2	0.16	1193	95.98
Squamous cell carcinoma	2	0.16	1195	96.14
Uterine leiomyosarcoma	2	0.16	1197	96.30
Bladder perforation	1	0.08	1198	96.38
Cholecystitis	1	0.08	1199	96.46
Dehydration	1	0.08	1200	96.54
Esophageal disease	1	0.08	1201	96.62
Facial cellulitis	1	0.08	1202	96.70
Forestomach disease	1	0.08	1203	96.78
Goiter	1	0.08	1204	96.86
Grayanotoxin	1	0.08	1205	96.94

Primary Diagnoses in Goats				
Diagnosis	Frequency	Percent	Cumulative Frequency	Cumulative Percent
Hepatic abscesses	1	0.08	1206	97.02
Hypocalcemia	1	0.08	1207	97.10
Injection reaction	1	0.08	1208	97.18
Keratitis	1	0.08	1209	97.26
Laminitis	1	0.08	1210	97.35
Lymphnodal abscesses	1	0.08	1211	97.43
Mammary sarcoma	1	0.08	1212	97.51
Melanoma	1	0.08	1213	97.59
Mesentery	1	0.08	1214	97.67
Muscle atrophy	1	0.08	1215	97.75
Myositis	1	0.08	1216	97.83
Nephrotoxicosis	1	0.08	1217	97.91
Oral disease	1	0.08	1218	97.99
Osteomyelitis	1	0.08	1219	98.07
Osteoporosis	1	0.08	1220	98.15
Otitis media	1	0.08	1221	98.23
Pnuemonia	1	0.08	1222	98.31
Portal vein hypoperfusion	1	0.08	1223	98.39
Pulmonary abscess	1	0.08	1224	98.47
Pulmonary hemorrhage	1	0.08	1225	98.55
Pyelonephritis	1	0.08	1226	98.63
Renal dysplasia	1	0.08	1227	98.71
Renal failure	1	0.08	1228	98.79
Renal necrosis	1	0.08	1229	98.87
Renal papillary necrosis	1	0.08	1230	98.95
Renal tubular necrosis	1	0.08	1231	99.03
Sarcoma	1	0.08	1232	99.12

Primary Diagnoses in Goats				
Diagnosis	Frequency	Percent	Cumulative Frequency	Cumulative Percent
Synovitis	1	0.08	1233	99.20
Thymic lymphoma	1	0.08	1234	99.28
Urethral infarct	1	0.08	1235	99.36
Urethral stricture	1	0.08	1236	99.44
Urethritis	1	0.08	1237	99.52
Urinary bladder perforation	1	0.08	1238	99.60
Uterine amyloidosis	1	0.08	1239	99.68
Uterine carcinoma	1	0.08	1240	99.76
Vesicopreputial anastomosis stricture	1	0.08	1241	99.84
White muscle disease	1	0.08	1242	99.92
Yew toxicity	1	0.08	1243	100.00
