# Supplementary material for: Effect of age, sex, and county on postmortem findings in goats and sheep in Tennessee (USA), 2017–2021
Source: PLoS One. 2024 Dec 13;19(12):e0315680. doi: 10.1371/journal.pone.0315680 (PMC11642948; doi:10.1371/journal.pone.0315680)
Supplement: S2 Table — (PDF) [file pone.0315680.s002.pdf]

S2 Table.

Primary Diagnoses in Sheep				
Primary Diagnosis	Frequency	Percent	Cumulative Frequency	Cumulative Percent
Endoparasitism	182	32.85	182	32.85
Abortion	77	13.90	259	46.75
No diagnosis	42	7.58	301	54.33
Pneumonia	42	7.58	343	61.91
Neurologic disease	30	5.42	373	67.33
Trauma	20	3.61	393	70.94
Intestinal	15	2.71	408	73.65
Copper toxicity	13	2.35	421	75.99
Forestomach disease	11	1.99	432	77.98
Emaciation	9	1.62	441	79.60
Sepsis	9	1.62	450	81.23
Anemia	6	1.08	456	82.31
Abomasal disease	5	0.90	461	83.21
Metritis	5	0.90	466	84.12
Urolithiasis	5	0.90	471	85.02
Caseous lymphadenitis	4	0.72	475	85.74
Dermatitis	4	0.72	479	86.46
Esophageal disease	4	0.72	483	87.18
Osteomyelitis	4	0.72	487	87.91
Dystocia	3	0.54	490	88.45
Heart failure	3	0.54	493	88.99
Mesentery	3	0.54	496	89.53
Nephritis	3	0.54	499	90.07
Oral	3	0.54	502	90.61

Primary Diagnoses in Sheep				
Primary Diagnosis	Frequency	Percent	Cumulative Frequency	Cumulative Percent
Pulmonary edema	3	0.54	505	91.16
Salmonellosis	3	0.54	508	91.70
Dystocia	3	0.54	511	92.24
BVDV	2	0.36	513	92.60
Copper deficiency	2	0.36	515	92.96
Copper toxicosis	2	0.36	517	93.32
Hepatic abscesses	2	0.36	519	93.68
Kidney disease	2	0.36	521	94.04
Mastitis	2	0.36	523	94.40
Nasal adenocarcinoma	2	0.36	525	94.77
Pregnancy toxemia	2	0.36	527	95.13
Abscess	1	0.18	528	95.31
Atlanto-occipital joint abscess	1	0.18	529	95.49
Bladder	1	0.18	530	95.67
Cellulitis	1	0.18	531	95.85
Copper Toxicity	1	0.18	532	96.03
Cryptorchidism	1	0.18	533	96.21
Ectoparasitism	1	0.18	534	96.39
Hepatic lipidosis	1	0.18	535	96.57
Hepatic necrosis	1	0.18	536	96.75
Hepatic trematodiasis	1	0.18	537	96.93
Hepatopathy	1	0.18	538	97.11
Hydrometra	1	0.18	539	97.29
Laryngotracheitis	1	0.18	540	97.47
Lymphoma	1	0.18	541	97.65

Primary Diagnoses in Sheep				
Primary Diagnosis	Frequency	Percent	Cumulative Frequency	Cumulative Percent
Mammary gland abscess	1	0.18	542	97.83
Nasal fistula	1	0.18	543	98.01
Osteoarthritis	1	0.18	544	98.19
Otitis media	1	0.18	545	98.38
Peritonitis	1	0.18	546	98.56
Pleuritis	1	0.18	547	98.74
Pregnancy Toxemia	1	0.18	548	98.92
Pulmonary dysmaturity	1	0.18	549	99.10
Renal Failure	1	0.18	550	99.28
Rhinitis	1	0.18	551	99.46
Skeletal muscle necrosis	1	0.18	552	99.64
Thymoma	1	0.18	553	99.82
White muscle disease	1	0.18	554	100.00
